# Experiences and needs of persons living with a household member infected with SARS-CoV-2: A mixed method study

**DOI:** 10.1371/journal.pone.0249391

**Published:** 2021-03-31

**Authors:** Janneke D. M. Verberk, Sibyl A. Anthierens, Sarah Tonkin-Crine, Herman Goossens, John Kinsman, Marieke L. A. de Hoog, Julia A. Bielicki, Patricia C. J. L. Bruijning-Verhagen, Nina H. Gobat

**Affiliations:** 1 Department of Medical Microbiology and Infection Prevention, University Medical Centre Utrecht, Utrecht, The Netherlands; 2 Julius Centre for Health Sciences and Primary Care, Department of Epidemiology, University Medical Centre Utrecht, Utrecht, The Netherlands; 3 Family Medicine and Population Health (FAMPOP), University of Antwerp, Antwerp, Belgium; 4 Nuffield Department of Primary Care Health Sciences, University of Oxford, Oxford, United Kingdom; 5 Health Protection Research Unit in Antimicrobial Resistance and Healthcare Associated Infections, University of Oxford, Oxford, United Kingdom; 6 Laboratory of Medical Microbiology, University of Antwerp, Antwerp, Belgium; 7 European Centre for Disease Prevention and Control, Solna, Sweden; 8 Infection Prevention and Control, University of Basel Children’s Hospital, Basel, Switzerland; 9 Paediatric Infectious Diseases Research Group, St George’s University of London, London, United Kingdom; University of Auckland, NEW ZEALAND

## Abstract

**Background:**

Households are important sites for transmission of SARS-CoV-2 and preventive measures are recommended. This study aimed to 1) investigate the impact of living with a person infected with SARS-CoV-2; 2) understand how household members implemented infection control recommendations in their home; and 3) identify the information and support needs of household members.

**Methods:**

For this observational mixed-methods study, households with a person with confirmed SARS-CoV-2 infection were recruited via drive-through testing sites of Municipal Health Services, healthcare worker screening or hospital emergency visits in the University Medical Centre Utrecht, the Netherlands and via primary care physicians, hospital emergency visits or preoperative screening in the University Hospital of Antwerp, Belgium. We recorded household characteristics, including characteristics of all household members, together with their views on prevention measures. In a subset of households one adult household member was asked to participate in an interview investigating their views on preventive measures. Survey data were analysed using descriptive statistics and interview data by rapid framework analysis. A triangulation protocol was used to integrate findings.

**Results:**

Thirty-four households (120 household members) were included in the quantitative survey. Twenty-two households were invited to be interviewed, of which 18 completed an interview (response 81.8%). Survey data showed that almost all households implemented some preventive measures, the use of face masks being least frequently reported. Measures taken depended on what was physically possible, the perceived severity of illness of the index patient and to what extent household members were willing to limit social interaction. Respondents did not believe in the effectiveness of wearing face masks within the house, and from the interviews this was explained by media coverage of face masks, impracticality and the stigma associated with wearing masks. Interviewees reported that quarantine had a high emotional burden and wished to have more information about the exact duration of quarantine, their own COVID-19 status, symptoms and when to seek medical help.

**Conclusion:**

People were willing to implement prevention measures, however actual adherence depended on perceived severity of illness and the perceived risk of becoming infected. Homes are social environments and recommendations for infection prevention should account for this context. Incorporating our findings into policy making could provide households with more relevant and actionable advice.

## Introduction

To preserve hospital capacity for severe COVID-19 patients, public health authorities in many parts of the world, including in Europe, have recommended that COVID-19 patients with mild infection be cared for at home. The World Health Organization (WHO) has provided guidelines for home care of patients with mild infection, adapted from those developed for Middle East Coronavirus (MERS-CoV) [1], and the European Centre for Disease Control (ECDC) further specified these with a focus on infection prevention and control in the home [2]. Given that the majority of patients with COVID-19 present with mild infection [3, 4], households are primary sites for treatment and care [5]. However, households are also important sites for disease transmission, including among asymptomatic and paucisymptomatic household members [6–9]. Many studies report COVID-19 household secondary attack rates of 15%-20% for those sharing a residential address with the index person, but estimates are highly variable ranging from 4% to 44% [10–15].

In Europe, national governments adopt different strategies related to home-based care. In the Netherlands (NL) and Belgium (BE), for example, guidelines for home care of people with COVID-19 required them to isolate until they are 24 hours free of symptoms and at least seven days post illness onset. COVID-19 is a notifiable disease and must be reported to the Public Health Institute who will contact the patient and start contact tracing. In the Netherlands, this is executed by Municipal Health Services (MHS) who also send information by email to the household with guidelines for the index person and a separate letter with guidelines for the household members. In Belgium municipal services only provide guidelines and information for the index person.

To prevent spread of infection within the home, household members are advised to adopt infection prevention and control practices that include: isolation of the index patient (where feasible), rigorous hand hygiene, respiratory etiquette, disinfection of surfaces and materials used by the index person, frequent ventilation of rooms and using separated toilets. While information provided by public health authorities on infection control is important for transmission prevention, information alone is generally insufficient for people to adhere to recommendations which require a substantial change in behaviour away from normal routines. Recommendations require people living in a household to adopt new behaviours and/or adjust everyday habits and routines within the home. The extent to which people comply with these adaptations is influenced by many factors, including their perception of infection risk, their beliefs about the effectiveness of advice provided, their access to necessary materials and social norms [16]. Emotion will also play a role in adherence to guidelines and advice. For COVID-19, household members are being asked to distance themselves from the person who is ill, at a time when a natural human response would be to express connection and care. The psychological effects of restrictive public health measures may also lead to increased stress and tension in the home, which can be exacerbated by concern about infection risk and additional caring responsibilities [17]. Household members will have their own responsibilities, for example education or employment, their own health needs and may have additional caring responsibilities for older adults or young children.

Once a member of a household is ill with COVID-19, all members of the household have an important role to slow disease transmission. Understanding the support needs of household members to do this requires closer understanding of their experiences. This study aimed to understand how household members implemented recommendations to prevent infection transmission in their home, the impact of living with COVID-19 and to identify their information and support needs.

## Methods

### Study design

We conducted a rapid mixed-methods, descriptive study that used a convergent parallel design [18]. This design was selected as a pragmatic and efficient approach to collect data rapidly using complementary methods that can yield important insights during a public health emergency [19]. This study was conducted as part of a prospective, observational, clinical study that aimed to understand household transmission and epidemiology of SARS-CoV-2 in three European countries (RECOVER Household Study). This study is reported acknowledging the Good Reporting of A Mixed Methods Study (GRAMMS) criteria [20].

### Quantitative methods

#### Sampling

We identified patients with a confirmed SARS-CoV-2 positive test result in the Netherlands via drive-through testing sites of the Municipal Health Services (MHS) ‘Gooi en Vechtstreek’, ‘Zuid-Holland Zuid’, ‘Midden Gelderland’ and ‘Gelderland Zuid’ (mildly symptomatic individuals), via healthcare worker (HCW) screening programs (mildly symptomatic individuals) or via emergency visits (moderate/severe cases) in University Medical Centre Utrecht. For Belgium, recruitment took place via primary care physicians (mildly symptomatic individuals), emergency visits (moderate/severe cases) or preoperative screening in University Hospital Antwerp (asymptomatic cases). Patients received an information flyer about the study and were asked to contact the study team within 24 hours of their positive test result if they and their household members were willing to participate.

#### Data collection

**Fig 1:**
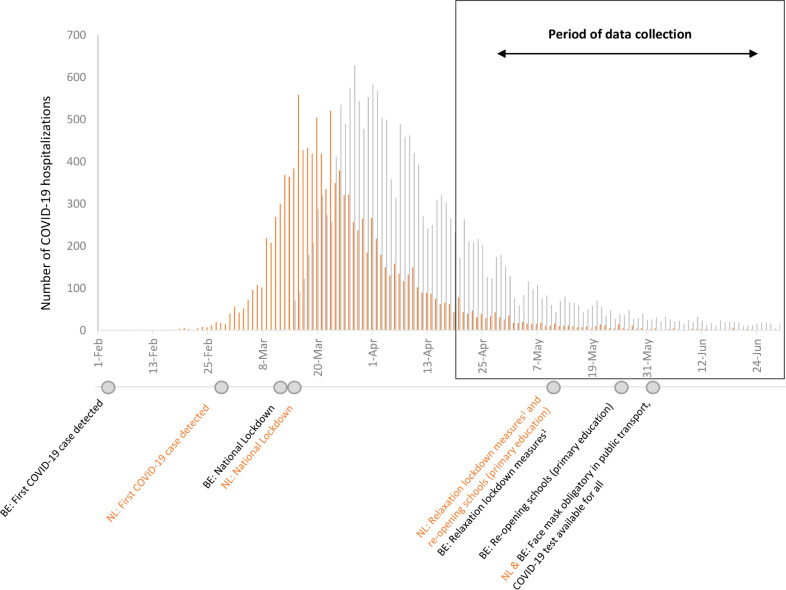
Number of COVID-19 hospitalizations in Belgium (BE) and the Netherlands (NL) including an overview of the national preventive measures taken during first epidemic wave, February-July 2020. Orange lines = the Netherlands, Black lines = Belgium. ^1.^One by one opening restaurants and bars, sporting clubs, sauna’s, upscaling public transport. Raw data obtained via National Public Health Institutes NL and BE: www.rivm.nl and www.sciensano.be.

For each household enrolled, a baseline questionnaire was completed by the household contact person containing questions regarding household characteristics and views on prevention measures (S1 File). From each household member, baseline characteristics were captured irrespective of symptoms. Thereafter, households were followed for at least 21 days and follow-up ended if no new disease episode emerged in the household. At the end of this follow-up, the main household contact person also completed a brief, validated index of wellbeing: the WHO (Five) Well-being Index (WHO-5) [21]. In the Netherlands, data collection started on the 20th of April and in Belgium on the 29th of April 2020 at a time where lockdown restrictions started being relaxed (see Fig 1). Data were collected via a specifically designed app and stored in an online secured web system. As these data were collected as part of the RECOVER Household Study, which is still running, we used the quantitative data collected to date.

#### Analysis

Data were analysed using descriptive statistics using Statistical Package for Social Sciences V.25.0.2 (SPSS, Chicago, Illinois, USA).

### Qualitative methods

#### Sampling

At enrolment, we invited every household member that was included in the study to participate in an interview. We interviewed an adult household member, who was not the index person, within 7–15 days after the COVID-19 diagnosis of the index case.

#### Data collection

From 5^th^ May to 9^th^ July 2020, two experienced qualitative researchers conducted telephone interviews in the local language using a semi-structured topic guide with open-ended questions (S2 File). Interviews covered the following topic areas: experience of home care, practices regarding transmission prevention, and the impact on daily life. Interviews were audio recorded with participants’ permission. We continued data collection until we reached saturation in order to formulate an answer to the research question.

#### Analysis

We conducted data collection and analysis concurrently. Researchers summarised data, including verbatim quotes, directly from the audio recording using an a priori framework that captured key areas of interest based on the research questions immediately after the interview was completed [19, 22]. Throughout the process, the team met weekly online to identify key patterns in the data and to compare and contrast emerging findings. Once data collection was complete, the research team reviewed and refined the key findings and their significance for policy recommendations. The team included senior social science academics, with expertise in sociology, psychology, behavioural science and public health emergency research, an infectious disease epidemiologist with expertise in public health and a social scientist working at European level on communicable disease prevention and control.

### Integration

We followed a triangulation protocol to integrate mixed-methods findings [23–25]. Following the separate analysis of qualitative and quantitative findings, key findings from both data sets were identified and listed. Quantitative findings were translated into qualitative statements, e.g. ‘95% of respondents indicated they felt calm and relaxed at least some of the time’. Each key finding was compared to all findings from the other dataset, developing a convergence coding matrix [23]. These paired comparisons were marked either as ‘Agreement’, ‘Partial agreement’ or ‘Disagreement’ [23]. If there were no data on a similar topic in the other dataset then the finding was marked as ‘Silence’.

### Ethics

Medical Ethical Committee Utrecht (NL) and Medical Ethics Committee UZA Antwerp (BE) provided review and ethical approval for this study (Reference number 20-185/D and 20/14/177 respectively). Written informed consent was obtained for each participant completing surveys. Verbal consent was obtained and audio recorded for each interview. All data were processed in accordance with the General Data Protection Regulation.

## Results

### Quantitative findings

As this research was part of an ongoing prospective study into household transmission of SARS-CoV-2, all 34 households that were enrolled up to mid July 2020 were included (NL: 22, BE: 12) (Fig 1). Baseline household characteristics are shown in Table 1. Households were mainly families, ranging from 2 to 9 members (median 3). Most households were recruited via HCW screening, resulting in index patients with mild COVID-19 disease; however, three patients were hospitalized during the study follow-up. Households included a total of 120 household members (86 adults and 34 children under 16 years of age) (Table 2).

**Table 1:** Baseline characteristics of households included in the study (n = 34).

Characteristics	
**Age category index case** (years, n, %)	
0–19	2 (5.9%)
20–39	17 (50.0%)
40–64	12 (35.3%)
65+	3 (8.8%)
**Severity of disease index case** (n, %)	
Mild	31 (91.2%)
Hospitalized	3 (8.8%)
**Household size** (persons, median, range)	3 (2–9)
**Household type** (n, %)	
Family	23 (67.7%)
Couple	7 (20.6%)
Friends	1 (2.9%)
Student accommodation	2 (5.9%)
Other	1 (2.9%)
**Number of bedrooms** (n, %)	
1	3 (8.8%)
2	3 (8.8%)
3	12 (35.3%)
>4	16 (47.1%)
**Sleeping in own bed** (n, %)	
Yes	22 (64.7%)
No	12 (35.3%)
**Index case shared usually bed with household member** (n, %)	
Yes	15 (44.1%)
No	19 (55.9%)
**Number of toilets in house** (n, %)	
1 toilet	8 (23.5%)
2 toilets	22 (64.7%)
>2 toilets	4 (11.8%)
**Presence of a sink in toilet room** (n, %)	
Yes, all toilets	25 (73.6%)
Yes, not all toilets	6 (17.6%)
No	3 (8.8%)
**Pets in household** (n, %)	
No	18 (52.9%)
Yes	16 (47.1%)
• Dogs	• 7 (43.8%)
• Cats	• 9 (56.3%)
• Rodents	• 1 (6.3%)
• Other pet	• 1 (6.3%)
**Frequency of visitors in household past 14 days** (n, %)	
Less than once a week	21 (61.8%)
1–3 times a week	9 (26.4%)
More than 3 times a week	4 (11.8%)

**Table 2:** Baseline characteristics of household members included in the study (n = 120).

Baseline characteristics	
**Sex** (n, %)	
Male	59 (49.2%)
Female	61 (50.8%)
**Age category** (years, n, %)	
0–2	7 (5.8%)
11-Mar	14 (11.7%)
17-Dec	14 (11.7%)
18–29	30 (25.0%)
30–39	13 (10.8%)
40–49	18 (15.0%)
50–59	17 (14.2%)
60+	6 (5.0%)
Missing	1 (0.8%)
**Country of Birth** (n, %)	
The Netherlands	69 (57.6%)
Belgium	46 (38.3%)
Japan	1 (0.8%)
Morocco	2 (1.7%)
Suriname	1 (0.8%)
Missing	1 (0.8%)
Educational level*^#^ (n, %)	
High	35 (40.7%)
Middle	22 (25.6%)
Low	28 (32.6%)
Missing	1 (1.2%)
Paid work* (n, %)	
No	21 (24.4%)
Yes	64 (74.4%)
• Healthcare/Patient care	27 (42.2%)
• Children day care	0 (0.0%)
• Primary, secondary school	2 (3.1%)
• Post-secondary school	2 (3.1%)
• Other	33 (51.6%)
Missing	1 (1.2%)

*asked to participants aged >16 (n = 86).

# Low = no education or primary education, Middle = Secondary education and Vocational secondary education, High = Higher Professional education, University

#### Access to materials to prevent transmission

**Fig 2:**
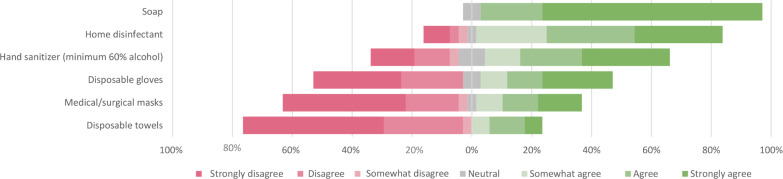
Access to materials over the past week to prevent spread of SARS-CoV-2 within the house as self-reported by each contact person of the household (n = 34).

The majority of households reported that they had enough soap, disinfectants and hand sanitizer (Fig 2). Most reported that they did not have access to medical face masks and disposable materials such as gloves and towels.

#### Beliefs about effectiveness of preventive measures

**Fig 3:**
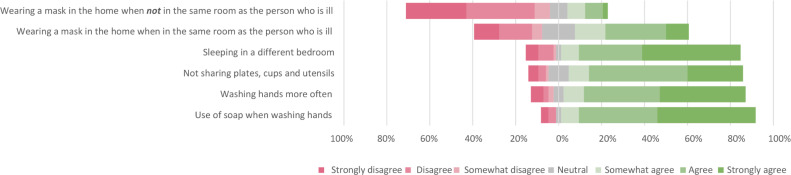
Beliefs of effectiveness of measures preventing spread of infection in households (n = 85).

Overall, the majority of household members were convinced about the preventive effect of frequent handwashing using soap, the use of separate plates, cups and utensils, and sleeping in different bedrooms than the person with COVID-19 (Fig 3). In contrast, the majority did not believe in the effectiveness of wearing face masks when the person with COVID-19 was not present. When the index case was present in the same room most people agreed masks were effective. For this last statement 15.3% answered neither agree nor disagree, this was higher than for other statements.

**Fig 4:**
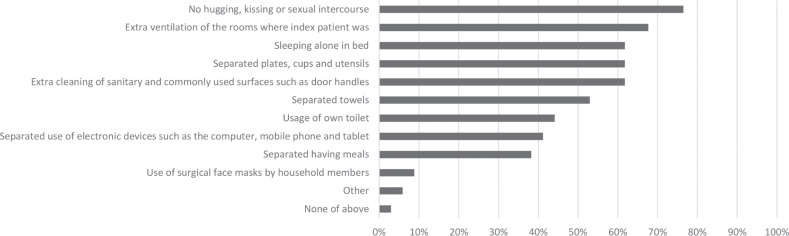
Overview of preventive measures taken in households to prevent transmission of COVID-19 as self-reported by each contact person of the household (n = 34).

Almost all households implemented some preventive measures: there was only one household whose members reported doing nothing (Fig 4). Most commonly households avoided physical contact and ensured extra ventilation of rooms. Use of face masks, segregated use of electronic devices and eating meals separately were least frequently reported.

#### Knowledge and information

Almost all (n = 80, 94.1%) respondents agreed that they knew where to find information about preventing the spread of infection in households. Seventy-seven percent agreed or strongly agreed that they had received enough advice: only nine (10.6%) respondents disagreed that they had received enough advice, four from Belgium and five from the Netherlands. Most respondents (n = 50, 58.8%) were confident that they could protect themselves from becoming infected, however answers varied: 9.4% strongly disagreed, 15.2% somewhat disagreed, 9.4% were neutral, 16.5% somewhat agreed, 31.2% agreed and 10.6% strongly agreed.

#### Wellbeing

**Fig 5:**
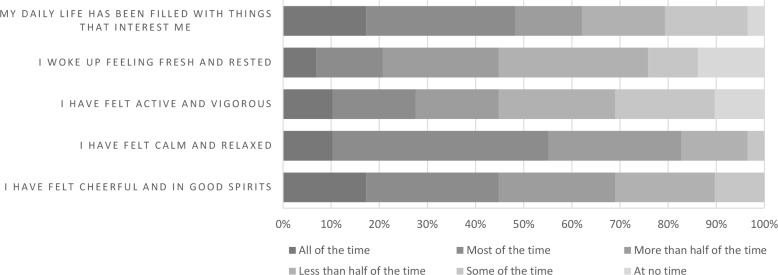
Overview of wellbeing at the end of follow-up as self-reported by each contact person of the household (n = 29).

At the end of the follow-up period, the contact person in the household filled in the WHO-5 (n = 29). The median index was 56 (range 16–100), with variation from respondents across the 5 statements (Fig 5). A score below 50 is an indicator to screen for depression.

### Qualitative findings

As qualitative data collection started later, 22 of the 34 households were invited for an interview. Of these, 18 (81.8%) responded positively and participated. Participants were mainly female (n = 10, 55.6%) with an age range from 25–77 (mean 43), 8 were from Belgium and 10 from the Netherlands. Interviews lasted between 20 to 45 minutes (mean 28 minutes).

#### Theme 1: Views on recommendations

*Implementing recommendations in the home*. Most participants reported that they ‘strictly follow the rules’ but adapted them to their own context and created their own reasoning for what they felt was effective. The physical environment influenced what households could feasibly implement. For example, people living in a one-bedroom flat could not comply with recommendations about separate sleeping arrangements. Hygiene measures like handwashing and disinfecting surfaces were reported to be easily implemented and followed up by most participants.

*“We all knew the rules that we should follow*, *and we really try and do our best*, *but in a household with 3 little children*, *it is not always possible to stick to it all the time*. *Hygiene measures were respected but face masks and self-isolation*, *that doesn’t work in our household*. *Also*, *the kids didn’t show any symptoms so we didn’t think it was necessary*.” *(Int1 BE)*

Isolation of the person who was ill was felt to be difficult for both severely ill as well as mild or asymptomatic people. For severely ill people, some participants reported that they wanted to keep a close eye on, and monitor, their loved ones in case their illness got worse, even though isolation in a separate room was possible.

*“One night she seemed to have difficulties breathing so I moved her downstairs to the living room as it is more airy there and I could sleep next to her so I would watch out for her in case it would get worse and then we could immediately go to the hospital when necessary*. *That was quite a difficult night*.” *(Int1 BE)*

For mild or asymptomatic illness, the need for physical isolation was difficult to accept as people were viewed as less contagious.

None of the participants wanted to wear face masks in their home as it was considered inconvenient and stigmatizing. They reported they would wear face masks outside the home.

“My daughter asked whether she would have to wear a face mask in the house, but I don’t want her to do that, I don’t want to give her the feeling that she is the ‘contagious’ person, I want her to feel normal.” (Int2 BE)

*“Wearing masks is not that comfortable especially when it is hot*, *but if it is only in situations where physical distancing cannot be respected it is important to wear them*.” *(Int1 BE)*

Respondents justified not wearing masks by indicating doubts about the effectiveness of masks. Some raised the fact that experts and health care professionals did not agree on the evidence of effectiveness, which had been extensively discussed in the media.

*Factors influencing transmission prevention behaviours*. How strict people were in following recommendations was linked to the perceived risk of becoming infected or the ‘seriousness’ of the infection.

*“You can try your best to keep your distance but it is very difficult to do that all the time in your home and especially if the person does not look that ill at all and seems very well*. *It gives you a double feeling*: *you know you should do it to prevent infection spreading*, *but the person seems fit and not ill at all*, *so it seems unnecessary*.” *(Int2 BE)*

In many households there were a few days between symptoms and a positive test result. Some interviewees thought that transmission would already have happened so they did not undertake preventive measures. A common coping strategy was accepting the possibility that all household members might become infected at some point.

*“My daughter just wants to get it as quickly as possible*, *as she says then I have had it and I don’t have to be so careful all the time*. *She is also convinced that the younger adults will have mild symptoms so they are not that anxious about it*.” *(Int2 BE)*

*“We are not taking any extra preventive measures now as the virus is already in the house and I think well if I have to get it I will get it and so be it*. *I just hope it won’t be too bad*.” *(Int8 BE)*

Testing was very important for participants as it gave them reassurance that they did not have the virus and motivated them to continue prevention measures.

The challenge of balancing preventive measures whilst simultaneously preserving a homelike environment was frequently highlighted. People found it particularly difficult to keep a physical distance from their children, especially if they were young. Even with older children people felt they still had a caring role and did not want to emotionally detach themselves by not physically comforting children when they were ill. Some mentioned that children did not understand what was happening so changes were hard to maintain.

“*As a mother you can’t put your daughter in self-isolation in her room all by herself*. *She would be miserable*, *no contact with your daughter*, *that is against my motherly instinct*. *I can’t make myself do that*.” *(Int2 BE)*

Interviewees reported that maintaining strict preventive measures over time was hard, and most of them indicated that after a few days, preventive measures were ‘taken with a grain of salt’, especially when the ill household member had mild symptoms.

#### Theme 2: Experiences of home care and impact on daily life

*Emotional burden*. The emotional impact of living with and caring for a relative with COVID-19 was significant and many different emotions were expressed. Participants reported feelings of helplessness, lack of control over the situation as well as acceptance and ‘taking it day by day’. Some were worried about being the source of transmission to others.

*“The moment the results come in and it is positive*, *it really feels like a shock*, *because then you really start to think we can all get infected now*. *I was hospitalized a few years ago with pneumonia and the thought I would have to go through that again and maybe being put in a coma*, *that gives me so much anxiety*.” *(Int2 BE)*

Many participants experienced an initial reaction of anger towards the ill person. Some were frustrated that they had to enter quarantine just as lockdown measures had relaxed, or because they had no clear idea on how long quarantine would be.

*“My first reaction to the positive test of my son was angriness because I felt that he could have gotten tested earlier on*. *He had already had loss of taste for over a week and that is one of the symptoms*, *and I had asked him to get tested*. *And now we all as a family have to go in quarantine because of it*. *I do realize it is not a nice feeling for him*, *but it has a big impact on all of us*, *my other daughter just started with an internship just after lockdown and now she can’t continue with it already because of this*.” *(Int4 BE)*

Despite this there were feelings of solidarity within the household and people reported ‘we are in it together’.

Direct care for the person with COVID-19 was often not needed or not seen as official ‘care’ for a patient. However, participants had concerns about their own health and the health of their loved ones. Some expressed having additional stress from having to monitor the patient to see whether symptoms were worsening. Interviewees indicated that they would prefer to have clear instructions on how to monitor their ill loved one, particularly regarding the need to seek medical assistance. Participants were concerned about sudden worsening of symptoms and practicalities like finding childcare if they needed to go to hospital.

*Stigma*. Participants discussed the stigma around a positive test and fear of reactions of other people who were scared of being infected themselves. Some felt others were judging them for not following the rules.

“*When I tell it [that my daughter has COVID-19] to other people*, *they see me as ‘contagious’ and they immediately respond with ‘oh*, *I hope I haven’t been too close to her’*. *Also they all reflect back on when they have last seen me and get panicky about it*, *it really is not a nice feeling*. *It reminds me of people who were having lepra*, *those people probably feel the same*. *Nobody really asks ‘hope you are well’ but they think about themselves first*. *It makes me a bit sad*.” *(Int2 BE)*

*“People are scared of you and your children*…*and*. . . .*scared to become infected*. . . .*I notice that I get emotional again because of this*, *sorry*. *That people don’t want to help [babysit while you have to bring your husband to the hospital] because they are afraid they will become infected*.” *(Int7 NL)*

*Coping with quarantine*. Participants understood the importance of implementing preventive measures at home and staying in quarantine. However, quarantine was experienced as a significant burden especially as lockdown measures had recently been relaxed. Many interviewees emphasized the significant impact the quarantine had on their mood; reporting feeling depressed, bored and having a lack of control.

*“You don’t have a choice*! *You are locked up with all household members*! *[…] The hardest thing is that you don’t know when your quarantine is over*.” *(Int6 NL)*

Household members reported that it was more difficult to be the person in the household who did *not* have COVID-19. Some felt it was easier to be diagnosed because then it was clear when you could go back to work. In the Netherlands and Belgium, household members should stay at home for at least 14 days after the resolution of symptoms in the last case in the household. For some household members, this meant that some were in quarantine for almost two months as quarantine duration was extended each time another household member developed symptoms and became ill.

*“Can I say something weird*? *Make sure you get the virus yourself*. *Then you have symptoms*, *have access to a test and if you don’t have symptoms anymore for 24 hours you are released*. *I am in quarantine for four weeks now because nobody trusts that I am corona-free*. *If I had corona I will be ill for a few days but thereafter at least I can go to work*.” *(Int1 NL)*

Despite these discussions household members did not indicate that they had stopped prevention measures in order to try and get infected as they were still uncertain about how COVID-19 would affect them. Interviewees all stressed the importance of staying positive and accepting the situation. Coping strategies included talking to family members and wider support networks, finding time for themselves to relax and making plans for when quarantine ended.

#### Theme 3: Information needs

The immediate provision of information from an official source was seen as an effective approach to meet household information needs.

*“From the moment it became clear that it was corona*, *the Municipal Health Service has sent such a package of information that you immediately know what and how to respond*.” *(Int7*, *NL)*

Although households received extensive information, many unanswered questions remained. Participants indicated they wanted more targeted information, specific to their needs. In Belgium, there was a wish for more information for other members of the household beyond information for the person who was ill. In both countries, interviewees expressed their concern about contradictory information and policies from different institutions. Guidelines from the national government, employers and schools were not always aligned and created confusion.

*“Guidelines of the hospital [employer] are different from national policy*. *That is confusing*…*who to trust*? *[*…*] So we each follow the policy of our own employer*, *and the children follow that of the Municipal Health Service*. *If I was tested negative*, *the Municipal Health Service said you have to stay home because of symptoms*, *but according to my employee I can go to work*. . . .” *(Int5 NL)*

Most household members were initially not aware of the quarantine rules for themselves. When the person with COVID-19 did not have symptoms anymore and was allowed to go outside other household members realised that they would have to be in quarantine for 14 more days. According to the interviewees, this should have been communicated more clearly at the beginning of the quarantine. Some reported they would have considered staying elsewhere.

Participants wanted information to be pertinent to their situation, taking into account their social environment but also the severity of illness. Therefore, according to interviewees, in Belgium, clearer information was needed regarding the duration of the symptoms, how long people are contagious and when to call a doctor or go to hospital.

*“I could really have used some more specific information*, *especially on the duration of self-isolation*. *You find a lot of contradictory information on the internet on that*, *but then when I had doubts I would call our GP*. *But I would like clear guidance on how long does the infected person have to stay in self-isolation*, *when can you say I am symptom free*, *what does that mean and how long are you contagious*?” *(Int5 BE)*

In both countries, participants wanted clear information on what they had to do in their households, preferably in the form of a checklist, but also information on how to adapt guidelines to their context. Lastly, participants mentioned the multiple sources of information which created information overload and confusion when sources did not agree.

### Integration

Key findings were identified for both datasets following the initial analyses. The quantitative analysis produced 26 key findings and the qualitative analysis produced 49. Sixty-six paired comparison were made of which 4 showed Agreement, 23 showed Partial Agreement, 4 showed Disagreement and 35 were marked as Silence. Silences indicated where questionnaires and interviews had purposely asked different questions and were therefore expected. The remaining comparisons were largely in agreement.

Partial agreement was more frequent than full, because qualitative data added detail to findings identified in the questionnaire and helped pick up nuances in participants’ experiences. Both datasets indicated that participants followed recommendations, however qualitative data were able to show where people were restricted in adherence due to their physical or social environment (e.g. one-bedroom flat, living with young children who didn’t understand changes) and that measures were difficult to maintain over time. The small number of households reporting face mask use in the survey was explained by participants’ accounts of masks being impractical and stigmatising. Both the questionnaire and interviews indicated uncertainty about the effectiveness of face masks. In the questionnaire only 31% of participants indicated confidence that they could protect themselves from the virus, interviews explained this as participants talked about the perceived inevitability of being infected once the virus was in their household. Both datasets indicated that participants were happy with information received although interviews identified that participants wanted tailored information.

There were four items showing disagreement, all of which linked to the WHO-5 and participant wellbeing. Questionnaires indicated that all participants felt cheerful and in good spirits at least some of the time and 95% indicated that their life was filled with things of interest. Interviews indicated greater struggles with well-being, with participants highlighting the burden of quarantine, uncertainty and anxiety about their own health and the health of loved ones. Interviews were also able to identify the coping strategies which participants used to manage their wellbeing and highlight areas for support.

## Discussion

In this study we aimed to understand and capture the experiences and needs of household members living and caring for a person infected with COVID-19. The key findings and policy suggestions derived from the results are described in Box 1. Survey data showed that almost all households implemented some preventive measures. Maintaining measures depended on the severity of illness, the perceived risk of getting infected and disruption to usual social interaction: this study provides a clear signal regarding views that wearing face masks within the house and isolating the COVID-19 diseased person in a separate room is undesirable.

**Box 1. Key messages and policy suggestions to optimise support for household members living with a person who has COVID-19**

- **Address illness severity in messaging:** Messaging related to preventing disease transmission in the home should address different illness severity of the index patient: i.e. regardless of how ill the patient appears, infection risk remains.
- **Consider testing all household members regardless of symptoms.** Having a negative test result while living with someone diagnosed with COVID-19 will motivate household members to maintain preventive measures within the home. Thereby, testing can shorten the duration of quarantine.
- **Motivate people to get tested immediately when symptoms start.** Explain the benefits to the whole household of people getting tested early and the need to implement stringent infection prevention measures as soon as concerns arise.
- **Emphasise the value of perseverance and changing habits for preventing infection in the home.** Messaging should emphasise that infection prevention is useful and important regardless of how long household member have been living with a person who is ill with COVID-19. Becoming infected isn’t inevitable: every effort counts.
- **Help household members provide quality care at home:** Information about how to care for household members with COVID-19 and when to seek medical care should be provided. Checklists are seen as particularly useful as a way of providing information.
- **Communicate directly with household members:** Information should be directed at household members and provided to them when they are living with a person who has COVID-19. Immediate provision of information from an official source is an effective approach to meet household member information needs.
- **Normalise emotional responses of household members to the index person:** anger, fear, anxiety and feeling overwhelmed are normal and legitimate emotions when a household member first becomes unwell. Encouraging households to discuss how they might feel if someone were to become unwell and a strategy for managing that scenario, can help build preparedness and resilience.
- **Share solutions that others have found to work.** The home is a social environment. Preventive measures have a greater chance to be adopted, maintained and successful if they work within a household’s daily rituals and routines.
- **Consistency is key:** Different policies and guidelines from schools, employers and national government create confusion.

Our study highlights the emotional impact quarantine has on individuals’ lives and supports findings from others who have conducted research in this area [17, 26]. People who are quarantined have a high level of distress and they may demonstrate post-traumatic stress symptoms when in quarantine for more than 10 days [17]. Reduced social and physical contact due to quarantine has also been associated with anxiety and anger up to 4–6 months after release [17]. Household members experience of stigma is also consistent with existing literature on the effects of isolation practices in patients with infectious diseases [26]. Lack of clear and consistent guidance about what actions to take during quarantine adds further stress and confusion [17]. In our study, participants were positive about the information they received, however they indicated that guidance on duration of quarantine from different sources were confusing. Clear information about the duration of quarantine and to whom it applies should be clear and specific to different contexts/groups [27]. Rapid testing after a week of quarantining and repeat testing the same day to identify false positive tests and avoid unnecessary quarantine for household members could be a solution [28].

Some preventive actions were easily implemented (e.g., washing hands) whereas others were less widely accepted (e.g. self-isolation, eating separately, wearing masks). Knowledge about the different extents to which behaviours are considered relevant, feasible and acceptable can help to inform communication and other interventions as a means of encouraging uptake of behaviours which are enacted less often and yet are considered essential for transmission prevention. Further, household members sometimes believed household transmission had already taken place as the index case waited too long for testing. Messaging to promote implementation of preventive measures at symptom onset, instead of waiting for a positive test result, are needed [29].

In this study there were four instances of disagreement between the survey and interview data, and they were all linked to the WHO-5. This may be explained by the fact that the WHO-5 index was filled out by only the contact person of the household at the end of follow-up–when in most cases quarantine was finished and COVID-19 in the household was over–while interviews were undertaken in ‘the heat of the moment’ and participants were experiencing greater disruption. We therefore expect that during the disease/quarantine period, WHO wellbeing scores may be lower than indicated here.

### Strengths and limitations

To the best of our knowledge, this is the first rapid mixed-methods study capturing experiences of prevention strategies in households during the first COVID-19 pandemic wave in Europe. Rapid qualitative methods are well suited to a public health emergency context [19]. A strength of our study was the team-based approach that included social science, epidemiology and policy perspectives that were important to shape and focus emerging findings to inform response actions. Interpretation of data was discussed iteratively within the multidisciplinary team resulting in new insights. A limitation of these methods is that they are subject to errors or misinterpretation, for example at the data summary stage, and bias. Full transcription of qualitative data would have extended time for data collection and analysis: in pandemic situations pragmatic decisions are often made whilst acknowledging limitations [30]. Capturing perceptions and experiences within context and during the first outbreak, with any associated uncertainty, will be highly informative and critical to developing more effective clinical and communication strategies, as well as acceptable and feasible public mitigations strategies.

We captured views of household members living in different types and sizes of homes and those living with people with variation in COVID-19 illness severity. However, our sample consisted mainly of highly-educated people aged 18–60 years. This can partly be explained by the sampling strategy we used (recruitment via HCW screening). People from other socio-economic backgrounds may have been impacted differently and may have different information needs. Nonetheless, some of our findings, particularly related to the emotional impact, will have salience for these groups. Views and experiences of younger and older age groups remained underexplored. Although saturation was achieved for qualitative data, at the time of conducting this study only 34 households were included in the quantitative survey. This low number restricted us to descriptive analysis but still gave us relevant information that we could triangulate with the qualitative data in order to have a fuller picture on the implemented prevention measures within households. Lastly, we captured data during the first pandemic wave in Europe. However, despite our best efforts to conduct rapid research, our data were collected after the peak; views and behaviour will change over time, and are influenced by the epidemic curve and national public health measures. We therefore recommend for future research to repeat this study during the peak of any future wave and compare the results.

## Conclusions

This study provided valuable insights about the preventive measures household members in the Netherlands and Belgium implemented during quarantine, when living with a SARS-CoV-2 positive household member. People were willing to implement some prevention measures, however adherence depended on the perceived severity of illness and the perceived risk of becoming infected. Not all recommendations were followed as they limited social interaction within the household, or because they went contrary to social norms and expectations. We therefore present policy suggestions, to acknowledge the context in which people live and to provide flexible guidance which can be implemented with minimal disruption, ensuring that transmission can be reduced as much as possible and considering the wellbeing of household members.

## Supporting information

S1 File Questionnaires. (PDF)

S2 File Interview topic guide. (PDF)

## References

[1] World Health Organization. Home care for patients with suspected or confirmed COVID-19 and management of their contacts—interim guidance. Geneve: WHO; 2020 [25 May 2020]. Available from: https://www.who.int/publications/i/item/home-care-for-patients-with-suspected-novel-coronavirus-(ncov)-infection-presenting-with-mild-symptoms-and-management-of-contacts.

[2] European Centre for Disease Prevention and Control. Infection prevention and control in the household management of people with suspected or confirmed coronavirus disease (COVID-19) Stockholm: ECDC; 2020 [25 May 2020]. Available from: https://www.ecdc.europa.eu/sites/default/files/documents/Home-care-of-COVID-19-patients-2020-03-31.pdf.

[3] World Health Organization. Coronavirus disease 2019 (COVID-19) situation report—46. Geneve: WHO; 2020 [17 July 2020]. Available from: https://www.who.int/docs/default-source/coronaviruse/situation-reports/20200306-sitrep-46-covid-19.pdf?sfvrsn=96b04adf_4.

[4] Ge H, Wang X, Yuan X, Xiao G, Wang C, Deng T, et al. The epidemiology and clinical information about COVID-19. European Journal of Clinical Microbiology & Infectious Diseases. 2020;39(6):1011–9. 10.1007/s10096-020-03874-z 32291542 PMC7154215

[5] Chan EYY, Gobat N, Kim JH, Newnham EA, Huang Z, Hung H, et al. Informal home care providers: the forgotten health-care workers during the COVID-19 pandemic. Lancet (London, England). 2020;395(10242):1957–9. 10.1016/S0140-6736(20)31254-X . 32497509 PMC7263813

[6] Jiang XL, Zhang XL, Zhao XN, Li CB, Lei J, Kou ZQ, et al. Transmission potential of asymptomatic and paucisymptomatic SARS-CoV-2 infections: a three-family cluster study in China. The Journal of infectious diseases. 2020. Epub 2020/04/23. 10.1093/infdis/jiaa206 32319519 PMC7188140

[7] Chan JF, Yuan S, Kok KH, To KK, Chu H, Yang J, et al. A familial cluster of pneumonia associated with the 2019 novel coronavirus indicating person-to-person transmission: a study of a family cluster. Lancet (London, England). 2020;395(10223):514–23. Epub 2020/01/28. 10.1016/S0140-6736(20)30154-9 31986261 PMC7159286

[8] Gao X, Yuan Z, Yang D, Li H, Zhang Y, Gao P, et al. A family cluster of severe acute respiratory syndrome coronavirus 2 infections. European journal of clinical microbiology & infectious diseases: official publication of the European Society of Clinical Microbiology. 2020. Epub 2020/04/10. 10.1007/s10096-020-03880-1 32270413 PMC7140591

[9] Huang R, Xia J, Chen Y, Shan C, Wu C. A family cluster of SARS-CoV-2 infection involving 11 patients in Nanjing, China. The Lancet Infectious diseases. 2020;20(5):534–5. Epub 2020/03/03. 10.1016/S1473-3099(20)30147-X 32119823 PMC7159019

[10] Jing Q-L, Liu M-J, Zhang Z-B, Fang L-Q, Yuan J, Zhang A-R, et al. Household secondary attack rate of COVID-19 and associated determinants in Guangzhou, China: a retrospective cohort study. The Lancet Infectious Diseases. 2020. 10.1016/S1473-3099(20)30471-0 32562601 PMC7529929

[11] Li W, Zhang B, Lu J, Liu S, Chang Z, Cao P, et al. The characteristics of household transmission of COVID-19. Clinical infectious diseases: an official publication of the Infectious Diseases Society of America. 2020. Epub 2020/04/18. 10.1093/cid/ciaa450 32301964 PMC7184465

[12] Cheng HY, Jian SW, Liu DP, Ng TC, Huang WT, Lin HH. Contact Tracing Assessment of COVID-19 Transmission Dynamics in Taiwan and Risk at Different Exposure Periods Before and After Symptom Onset. JAMA Intern Med. 2020. Epub 2020/05/02. 10.1001/jamainternmed.2020.2020 32356867 PMC7195694

[13] Madewell ZJ, Yang Y, Longini IM Jr., Halloran ME, Dean NE. Household transmission of SARS-CoV-2: a systematic review and meta-analysis of secondary attack rate. medRxiv. 2020:2020.07.29.20164590. 10.1101/2020.07.29.20164590 PMC7402051. 33315116 PMC7737089

[14] Wang Z, Ma W, Zheng X, Wu G, Zhang R. Household transmission of SARS-CoV-2. The Journal of infection. 2020;81(1):179–82. Epub 2020/04/10. 10.1016/j.jinf.2020.03.040 . 32283139 PMC7151261

[15] Rosenberg ES, Dufort EM, Blog DS, Hall EW, Hoefer D, Backenson BP, et al. COVID-19 Testing, Epidemic Features, Hospital Outcomes, and Household Prevalence, New York State-March 2020. Clinical infectious diseases: an official publication of the Infectious Diseases Society of America. 2020:ciaa549. 10.1093/cid/ciaa549 . 32382743 PMC7239264

[16] Bavel JJV, Baicker K, Boggio PS, Capraro V, Cichocka A, Cikara M, et al. Using social and behavioural science to support COVID-19 pandemic response. Nature Human Behaviour. 2020;4(5):460–71. 10.1038/s41562-020-0884-z 32355299

[17] Brooks SK, Webster RK, Smith LE, Woodland L, Wessely S, Greenberg N, et al. The psychological impact of quarantine and how to reduce it: rapid review of the evidence. The Lancet. 2020;395(10227):912–20. 10.1016/S0140-6736(20)30460-8 32112714 PMC7158942

[18] Schoonenboom J, Johnson RB. How to Construct a Mixed Methods Research Design. Kolner Z Soz Sozpsychol. 2017;69(Suppl 2):107–31. Epub 2017/10/11. 10.1007/s11577-017-0454-1 28989188 PMC5602001

[19] Johnson GA, Vindrola-Padros C. Rapid qualitative research methods during complex health emergencies: A systematic review of the literature. Social Science & Medicine. 2017;189:63–75. 10.1016/j.socscimed.2017.07.029 28787628

[20] O’Cathain A, Murphy E, Nicholl J. The quality of mixed methods studies in health services research. J Health Serv Res Policy. 2008;13(2):92–8. Epub 2008/04/18. 10.1258/jhsrp.2007.007074 . 18416914

[21] World Health Organization. Wellbeing Measures in Primary Health Care/The Depcare Project. Copenhagen: WHO 1998 [17 August 2020]. Available from: https://www.euro.who.int/__data/assets/pdf_file/0016/130750/E60246.pdf.

[22] Taylor B, Henshall C, Kenyon S, Litchfield I, Greenfield S. Can rapid approaches to qualitative analysis deliver timely, valid findings to clinical leaders? A mixed methods study comparing rapid and thematic analysis. BMJ Open. 2018;8(10):e019993. 10.1136/bmjopen-2017-019993 30297341 PMC6194404

[23] Farmer T, Robinson K, Elliott SJ, Eyles J. Developing and implementing a triangulation protocol for qualitative health research. Qual Health Res. 2006;16(3):377–94. Epub 2006/02/02. 10.1177/1049732305285708 . 16449687

[24] O’Cathain A, Murphy E, Nicholl J. Three techniques for integrating data in mixed methods studies. BMJ (Clinical research ed). 2010;341:c4587. Epub 2010/09/21. 10.1136/bmj.c4587 . 20851841

[25] Tonkin-Crine S, Anthierens S, Hood K, Yardley L, Cals JW, Francis NA, et al. Discrepancies between qualitative and quantitative evaluation of randomised controlled trial results: achieving clarity through mixed methods triangulation. Implement Sci. 2016;11:66. Epub 2016/05/14. 10.1186/s13012-016-0436-0 27175799 PMC4866290

[26] Cava MA, Fay KE, Beanlands HJ, McCay EA, Wignall R. The experience of quarantine for individuals affected by SARS in Toronto. Public Health Nurs. 2005;22(5):398–406. Epub 2005/10/19. 10.1111/j.0737-1209.2005.220504.x . 16229732

[27] Nasir L. The Checklist Manifesto: How to Get Things Right. London J Prim Care (Abingdon). 2010;3(2):124–. PMC3960713.

[28] Peto J, Carpenter J, Smith JD, Duffy S, Houlston R, Hunter DJ, et al. Weekly COVID-19 testing with household quarantine and contact tracing is feasible and would probably end the epidemic. *R Soc Open Sci*. (7):200915. 10.1098/rsos.200915 32742705 PMC7353981

[29] Wang Y, Tian H, Zhang L, Zhang M, Guo D, Wu W, et al. Reduction of secondary transmission of SARS-CoV-2 in households by face mask use, disinfection and social distancing: a cohort study in Beijing, China. BMJ Glob Health. 2020;5(5):e002794. 10.1136/bmjgh-2020-002794 . 32467353 PMC7264640

[30] Rutter H, Wolpert M, Greenhalgh T. Managing uncertainty in the covid-19 era 2020 [29 July 2020]. Available from: https://blogs.bmj.com/bmj/2020/07/22/managing-uncertainty-in-the-covid-19-era/. 10.1136/bmj.m3349 32873549

