# Supplementary material for: Experiences and needs of persons living with a household member infected with SARS-CoV-2: A mixed method study
Source: PLoS One. 2021 Mar 31;16(3):e0249391. doi: 10.1371/journal.pone.0249391 (PMC8011759; doi:10.1371/journal.pone.0249391)
Supplement: S1 File — (PDF) [file pone.0249391.s001.pdf]

S1. File. Questionnaires

Baseline household questionnaire, only filled in by the head of the household

NR.	BASELINE HOUSEHOLD QUESTIONNAIRE	ANSWER OPTION
1	Number of people within the household?	Number
2	What is the type of the household?	couple/family/student accommodation/friends/other
3	Description of your household?	Open text field
4	How many bedrooms do you have within your house?	Number
5	Does everybody sleep in their own bed?	Yes / No
6	How many people within the household share a bed?	Number
7	Did the index case shared a bed with a household member in the past two weeks?	Yes / No
8	Number of toilets in the house?	1/2/more than 2
9	Presence of a sink within the toilet room?	Yes, all toilets/Yes, but not all toilets/No
10	Do you have pets? (more answers possible)	No/Dog/Cat/Rodents/Other
11	In the past 14 days, how often did you or someone in your household receive visitors in the home?	less than once a week/1-3 times a week/more than 3 times a week
12	Over the past week there have been enough supplies of the following materials in my home to prevent spread of coronavirus infection:	
	• Hand sanitizer (minimum 60% alcohol)	Strongly disagree- disagree-somewhat disagree –neither agree nor disagree – somewhat agree – agree – strongly agree
	• Soap	Strongly disagree- disagree-somewhat disagree –neither agree nor disagree – somewhat agree – agree – strongly agree

	• Home disinfectant	Strongly disagree- disagree-somewhat disagree –neither agree nor disagree – somewhat agree – agree – strongly agree
	• Disposable towels	Strongly disagree- disagree-somewhat disagree –neither agree nor disagree – somewhat agree – agree – strongly agree
	• Medical or surgical masks	Strongly disagree- disagree-somewhat disagree –neither agree nor disagree – somewhat agree – agree – strongly agree
	• Disposable gloves	Strongly disagree- disagree-somewhat disagree –neither agree nor disagree – somewhat agree – agree – strongly agree
13	What extra protective measures have been taken to protect yourself and your housemates against transmission from your sick housemate (multiple answers possible)?	Sleeping in bed alone / no hugging, kissing or intercourse / use separate cutlery, cups, glasses / use separate meals / separate towels / extra cleaning of sanitary facilities and frequently used surfaces such as door handles / separate use of devices such as computer, mobile and Ipad / use of your own toilet / use surgical mask by housemates / extra ventilation of the room where your sick housemate was / other / none of the above

Baseline questionnaire, filled in by all adults (above 16 years) within the household

NR.	BASELINE PARTICIPANT QUESTIONNAIRE ADULT	ANSWER OPTION
1	What is your country of birth?	Open text field
2	Do you have payed work at this moment?	Yes / No
3	Do you work in (more answers possible)?	Patient care/ childcare / primary or secondary education / higher education / none of them
4	Are you a pupil/student?	Yes / No
5	In the past 2 weeks, did you left the home for work, traveling in public transport or socializing?	Yes / No
6	Have you been abroad in the past 2 weeks?	Yes / No
7	Which countries did you visit?	Open text field
8	What is the highest level of education that you have completed with a diploma?	For the Netherlands:<br>VMBO, MAVO, LBO/HAVO/VWO/MBO, MTS/HBO, university)/no education completed<br><br>For Belgium:<br>lager onderwijs/beroepssecundair onderwijs (BSO)/technisch secundair onderwijs (TSO)/algemeen secundair onderwijs (ASO)/hoger onderwijs/no education completed
9	What is your height in meters?	Open text field
10	What is your weight in kilograms?	Open text field
11	When was the last time you had symptoms of your upper airways with a nose cold and cough, possibly accompanied by a fever?	At this moment / less than 2 weeks ago / between 2 and 4 weeks ago / more than 4 weeks ago
12	Do you have any of the following chronic conditions, diagnosed by a medical doctor (multiple answers possible)?	Heart disease / lung disease (e.g. asthma or COPD) / immune disorder / diabetes / rheumatism / none

13	Are you currently taking doctor-prescribed medications for your chronic condition?	Yes / No
14	Which medicines do you use for your chronic condition?	Open text field
15	Are you currently being treated for cancer?	Yes / No
16	Are you currently taking any medications prescribed by your doctor to treat your cancer?	Yes / No
17	What medications are you using to treat your cancer?	Open text field
18	Do you have direct care responsibilities for your housemate who has the coronavirus infection?	No / Yes / I am the ill housemate
	**Please indicate the extent to which you agree with the following:**	
19	I know where to find information about preventing the spread of infection in my home when living with a person who is ill with the new coronavirus	Strongly disagree / disagree / somewhat disagree / neither agree nor disagree / somewhat agree / agree / strongly agree
20	I have received enough advice about what to do to prevent the spread of infection while living with someone who is ill with the new coronavirus	Strongly disagree / disagree / somewhat disagree / neither agree nor disagree / somewhat agree / agree / strongly agree
21	I am confident I can protect myself from becoming infected with the new coronavirus	Strongly disagree / disagree / somewhat disagree / neither agree nor disagree / somewhat agree / agree / strongly agree
22	I believe that the following recommendations will prevent spread of infection in my household:	
	• Washing hands more often	Strongly disagree / disagree / somewhat disagree / neither agree nor disagree / somewhat agree / agree / strongly agree
	• Using soap when washing hands	Strongly disagree / disagree / somewhat disagree / neither agree

		nor disagree / somewhat agree / agree / strongly agree
	• Not sharing plates, cups and utensils	Strongly disagree / disagree / somewhat disagree / neither agree nor disagree / somewhat agree / agree / strongly agree
	• Wearing a mask in the home when in the same room as the person who is ill	Strongly disagree / disagree / somewhat disagree / neither agree nor disagree / somewhat agree / agree / strongly agree
	• Wearing a mask in the home when **not** in the same room as the person who is ill	Strongly disagree / disagree / somewhat disagree / neither agree nor disagree / somewhat agree / agree / strongly agree
	• Sleeping in a different bedroom to the person who is ill	Strongly disagree / disagree / somewhat disagree / neither agree nor disagree / somewhat agree / agree / strongly agree

Baseline child (0-15 year) questionnaire, filled in by the head of the household

NR.	BASELINE PARTICIPANT QUESTIONNAIRE CHILD	ANSWER OPTION
1	In which country was your child born?	Open text field
2	Has your child been abroad in the past 2 weeks?	Yes / No
3	Which country-(ies) did your child visit?	Open text field
4	Has your child made use of (emergency) care at school / after-school care / child daycare in the past 2 weeks?	Yes / No
5	What is the height in meters of your child?	Open text field
6	What is the weight in kilograms of your child?	Open text field
7	When was the last time your child experienced symptoms of the upper airways with a nose cold and cough, possibly accompanied by a fever?	At this moment / less than 2 weeks ago / between 2 and 4 weeks ago / more than 4 weeks ago
8	Does your child have any of the following chronic conditions, diagnosed by a medical doctor (multiple answers possible)?	Heart disease / lung disease (e.g. asthma or COPD) / immune disorder / diabetes / rheumatism / none
9	Is your child currently taking any medications prescribed by the doctor for his / her chronic condition?	Yes / No
10	Which medicines does your child use for the chronic condition?	Open text field
11	Is your child currently being treated for cancer?	Yes / No
12	Is your child currently taking any medications prescribed by the doctor to treat the cancer?	Yes / No
13	What medications is your child taking to treat the cancer?	Open text field

End of study questionnaire, filled in by the head of the household

NR	STUDY END QUESTIONNAIRE ACCOUNT MEMBER	ANSWER OPTION
1	How many additional people in your household were diagnosed with a coronavirus infection during the study?	Number
	**During the follow-up of the household study:**	
2	I have felt cheerful and in good spirits	all of the time/most of the time/more than half of the time/less than half of the time/some of the time/at no time
3	I have felt calm and relaxed	all of the time/most of the time/more than half of the time/less than half of the time/some of the time/at no time
4	I have felt active and vigorous	all of the time/most of the time/more than half of the time/less than half of the time/some of the time/at no time
5	I woke up feeling fresh and rested	all of the time/most of the time/more than half of the time/less than half of the time/some of the time/at no time
6	My daily life has been filled with things that interest me	all of the time/most of the time/more than half of the time/less than half of the time/some of the time/at no time
7	Have you or anyone in your household encountered any technical difficulties using the App?	Yes/No
8	What problems have you and/or your housemates experienced?	Open text field
