# Supplementary material for: Experiences and needs of persons living with a household member infected with SARS-CoV-2: A mixed method study
Source: PLoS One. 2021 Mar 31;16(3):e0249391. doi: 10.1371/journal.pone.0249391 (PMC8011759; doi:10.1371/journal.pone.0249391)
Supplement: S2 File — (PDF) [file pone.0249391.s002.pdf]

S2 File: Interview topic guide

INTERVIEW QUESTIONS

Tell me a bit about yourself and your household
- Probes: what is your relationship with the patient? Who else lives in your household? (e.g. children, elderly relatives) Any pets?

Experience of home care
What was it like living with a person who was ill with the new coronavirus?
- Probes: what were the challenges? What worked well? What was your reaction when the patient was first diagnosed? What concerns did you have for the person, yourself, others living in the home?

Practices regarding transmission prevention
What did you do to try to avoid you or others living in the home becoming infected?
- Probes: what were the challenges trying to follow advice that was given?

What information did you receive about preventing others in the home becoming infected with COVID-19?
- Probes: From where did you get information? What kinds of questions did you have during the time you were living with the patient? Where did you look for answers to those questions? How easy was it to find the information you needed?
- What information would you have liked to have at the start to better prepare you for sharing a home with someone who is ill with COVID-19?

What sorts of things did you do at home to avoid becoming infected yourself?
- Probes: how did you set up the physical environment (e.g. move to a separate room)? Were you able to not share a room? How did you manage sharing of bathrooms, kitchens, and recreational space?
- Views on mask use: what experience if any, do you have of wearing masks? Probes: what kind of masks (cloth, medical etc), what is their view on masks, how do they think masks work, how do they feel wearing masks?

Impact on lives and livelihood
What responsibilities did you personally have regarding caring for the person who was ill?
- Probes: who cared for the person who was ill (if anyone)? What kinds of activities did you do for this person? E.g. cooking, cleaning of the room they were staying in etc. To what extent did this involve physical contact or close proximity?

How did you care for yourself during this time? Where do you get support?

How did your experience of living with a person ill with the new coronavirus impact your life? (i.e. beyond the impact of the coronavirus pandemic)
- Probes: What other caring responsibilities did you have? (e.g. children, elderly relatives). How did you balance these responsibilities? How did you balance demands related to work? What was the impact of working/ not working during this?

What was the hardest thing you and your family have had to deal with related to COVID-19? What did you do to cope over this time? What would have helped you cope better?**

Closure
What advice would you give to others who are living with a patient who is ill with the new coronavirus?**

Anything else you would like to share with us about your experience? Thank you for taking part in this research.
